# NIR-II Nanoprobes: A Review of Components-Based Approaches to Next-Generation Bioimaging Probes

**DOI:** 10.3390/bioengineering10080954

**Published:** 2023-08-11

**Authors:** Bryce Dunn, Marzieh Hanafi, John Hummel, John R. Cressman, Rémi Veneziano, Parag V. Chitnis

**Affiliations:** 1 Department of Bioengineering, George Mason University, Fairfax, VA 22030, USArvenezia@gmu.edu (R.V.); 2 Department of Physics, George Mason University, Fairfax, VA 22030, USA

**Keywords:** fluorescence, photoacoustic, imaging, nanoprobe, NIR-II, emission

## Abstract

Fluorescence and photoacoustic imaging techniques offer valuable insights into cell- and tissue-level processes. However, these optical imaging modalities are limited by scattering and absorption in tissue, resulting in the low-depth penetration of imaging. Contrast-enhanced imaging in the near-infrared window improves imaging penetration by taking advantage of reduced autofluorescence and scattering effects. Current contrast agents for fluorescence and photoacoustic imaging face several limitations from photostability and targeting specificity, highlighting the need for a novel imaging probe development. This review covers a broad range of near-infrared fluorescent and photoacoustic contrast agents, including organic dyes, polymers, and metallic nanostructures, focusing on their optical properties and applications in cellular and animal imaging. Similarly, we explore encapsulation and functionalization technologies toward building targeted, nanoscale imaging probes. Bioimaging applications such as angiography, tumor imaging, and the tracking of specific cell types are discussed. This review sheds light on recent advancements in fluorescent and photoacoustic nanoprobes in the near-infrared window. It serves as a valuable resource for researchers working in fields of biomedical imaging and nanotechnology, facilitating the development of innovative nanoprobes for improved diagnostic approaches in preclinical healthcare.

## 1. Introduction

**Figure 1:**
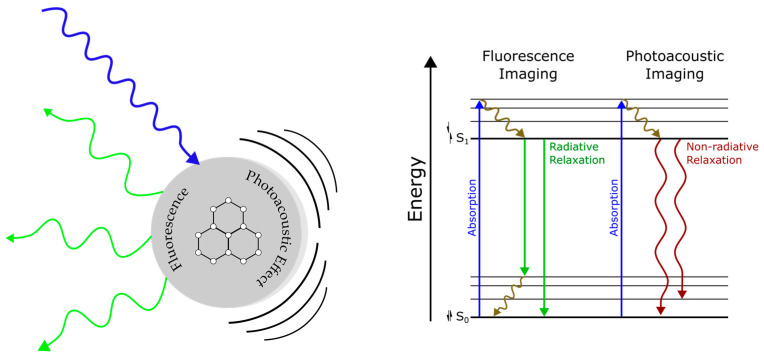
(**Left**) Illustration of fluorophore photoacoustic and fluorescence emission following light excitation; (**Right**) Jablonski diagram describing the energy acquisition and release pathways of fluorescence and photoacoustic imaging.

Fluorescence and photoacoustic (PA) imaging are two distinct but related optical methods for visualizing cells and tissue. Fluorescence imaging is based on the use of targeted tracers that absorb light energy and re-emit light of a different wavelength [1]. Fluorescence-based imaging is common practice in medical imaging and studies of biological systems for its high sensitivity and specificity [2]. PA imaging is a hybrid modality that leverages the advantages of optical and ultrasound imaging by exciting targets with pulses of laser light, which generate acoustic waves that are detected via ultrasound transduction. The low scattering of acoustic waves in tissue allows for high resolution imaging and deep tissue penetration. There exists a significant overlap between fluorescent and PA contrast agents due to their shared characteristic of light absorption. The complementary nature of PA and fluorescence emissions in response to light excitation is illustrated in Figure 1. Differences in signal transduction arise from radiative and non-radiative emission events. Both fluorescence and PA imaging are noninvasive, non-ionizing, capable of real-time imaging, and are often used together to provide complementary information about biological samples [3]. Researchers are actively exploring the design of nano-constructs with enhanced imaging properties in the near-infrared window.

The advantages of fluorescence and PA imaging are further enhanced using near-infrared II (NIR-II, 1000–2000 nm) light. NIR-II, also known as short-wave infrared (SWIR), has been demonstrated to achieve tissue imaging at a higher depth and lower auto-fluorescence and light scattering effects than lower-wavelength imaging windows. NIR-I (700–1000 nm), NIR-II (1000–2000 nm), and NIR-III (2000–2400 nm) are different near-infrared (NIR) imaging windows, each characterized by specific wavelength ranges and distinct applications in biomedical imaging [4]. The NIR-II window can further be divided into NIR-IIa (1300–1400 nm) and NIR-IIb (1500–1700 nm). Water exhibits a strong peak absorption maximum at 1450 nm, preventing fluorescence from 1400 to 1500 nm in biological samples. Although fluorophores that emit in the NIR-IIb window are scarce, this spectral range affords a higher resolution at sub-centimeter tissue depths due to a lower light scattering than NIR-IIa and lower absorption effects from endogenous chromophores. The limitations of existing contrast agents underscore the need for NIR-II probes for cell-specific imaging. 

This review article provides an up-to-date overview of the current state of research on NIR-II nanoprobes for fluorescence and photoacoustic imaging, highlighting recent advances, as well as the use of targeted agents for cell-specific imaging. NIR-II nanoprobes are a class of imaging agents designed to function in the near-infrared-II (NIR II) window (1000–1700 nm) and exhibit unique optical properties that enable them to overcome limitations observed in traditional NIR-I and visible-light imaging probes. Their distinct features and imaging capabilities make them valuable tools in fluorescence and photoacoustic imaging. This review covers a range of nanoprobes that demonstrate either NIR-II fluorescence, photoacoustic properties under NIR-II excitation, or possess both capabilities. The main components of NIR-II imaging probes are described: fluorophores, delivery technology, and targeting conjugates. Two main families of contrast agents, organic and inorganic, will be discussed. Transgenic PA and fluorescence contrast agents will not be discussed in this paper. Applications outside of PA and fluorescence imaging will not be discussed. Important considerations for probe construction are pharmacokinetic properties and the method of clearance [5]. Understanding these will lead to advancements in novel components and combinations thereof.

### Förster Resonance Energy Transfer

Förster Resonance Energy Transfer (FRET)-based processes are used extensively in medical imaging [6]. FRET is the process by which non-radiative energy is transferred from an excited donor (D) chromophore and an acceptor (A) chromophore by means of dipole–dipole coupling [7]. With the energy absorbed from the donor, the acceptor may itself fluoresce or quench by means of non-radiative decay. This process is highly dependent on the distance between the donor and acceptor, which is typically between 10 and 100 Å. FRET can be used to create new types of NIR-II imaging probes by creating conjugates of donor and acceptor molecules that are specifically targeted to a biological structure.

This process of intramolecular electron donation and acceptance has led to the rational design of novel fluorescence probes with red-shifted emission spectra into the NIR-II. The donor–π–acceptor (D–π–A) class of fluorophores has attracted recent attention for fluorescence in the NIR region [8]. The π-conjugated linker system between the donor and acceptor moieties enables an intramolecular charge transfer that may be tuned to NIR emission profiles. The factors affecting D–π–A systems are the strength of the donor and acceptor units, the length and composition of the π-linker system, and the planarization degree of the fluorophore. The change in FRET efficiency can be detected and used as a signal to image the target with high sensitivity.

## 2. Properties of NIR-II Fluorescence and Photoacoustic Imaging and Contrast Agents

Fluorescence imaging relies on the emission of fluorescent light by fluorophores when excited by a specific wavelength of light. The process involves the absorption of photons at a higher energy level, followed by the subsequent emission of photons at a lower energy level. This emission occurs within the NIR-II spectral range, making it suitable for in vivo imaging. Fluorescence imaging offers high sensitivity and excellent spatial resolution, enabling the detection of specific targets labeled with fluorescent probes. Photoacoustic imaging (PAI) combines the benefits of optical and ultrasound imaging. It relies on the photoacoustic effect, where short laser pulses are used to illuminate tissue or samples. The absorbed light energy induces a transient thermoelastic expansion, generating ultrasound waves that are detected by an ultrasound transducer. These ultrasound waves are then converted into images, providing information on the optical absorption properties of the tissue. PAI offers a deeper penetration than pure optical techniques. Combining the two modalities provides complementary information; fluorescence imaging excels at detecting specific molecular targets, while PAI enhances the visualization of tissue structures at depth. Additionally, both modalities can allow for simultaneous functional and molecular imaging to provide information about biological processes in real time. 

Optimal fluorochromes are characterized by several key photophysical properties. A high quantum yield (Φ_fl_, QY) indicates a greater efficiency in converting absorbed photons into fluorescent photons [9]. The molar extinction coefficient (ε) is directly related to the brightness of a fluorochrome, as it reflects the ability to absorb photons and emit fluorescence [10]. Understanding the fluorescence imaging depth is crucial for selecting the most suitable fluorochrome, as it determines the distance a photon can travel in a sample before it is randomly scattered or absorbed. This distance is known as the mean free path of the photon, and once this distance is exceeded, the clarity of the image is affected [11].

The absorbance cross-sections (ACS) for one-photon $\left(\sigma \right)$ and two-photon $\left(\delta \right)$ excitation govern the photon absorbance spectra of fluorochromes. In one-photon absorbance, a single photon is required to generate an excited state, while two-photon absorbance involves the simultaneous absorption of two photons to produce the same excited state. Both cross-sections are related to the beam intensity incident on the sample [12]. The probabilities of one and two photon absorbance are proportional to the beam intensity and square of beam intensity, respectively. Thus, two photon ACS is significantly smaller than one photon ACS making it a more attractive technique for precise excitation of a sample [12,13,14]. A narrow spectral bandwidth, high photostability under irradiation, water solubility, and small size are also desirable for a bioimaging fluorochrome [15,16]. The quantum yield, emission spectra, and bioimaging applications of major categories of organic fluorophores are given in Table 1. Likewise, the same properties for inorganic fluorophores can be found in Table 2.

Like fluorochromes for fluorescence imaging, the ideal PA contrast agent will exhibit a large molar extinction coefficient, a sharp-peaked absorption spectrum, peak absorption in the SWIR window, and high photostability. However, unlike fluorochromes, ideal PAI signaling compounds must exhibit a low quantum yield to efficiently convert heat energy to acoustic waves [17]. This efficient thermal conversion in addition to a strong NIR extinction coefficient is required for a strong PA signal. The pressure of the initial PA wave is dependent on four parameters: the Grunesian coefficient, the optical absorption coefficient, the heat conversion efficiency, and optical fluence, which are each linearly proportional to the initial pressure.

**Table 1:** Photophysical properties and applications of major organic NIR-II fluorophores.

Organic Fluorophore	Quantum Yield	Peak Excitation Wavelength (nm)	NIR-II Emission Wavelength (nm)	Bioimaging Application	Reference
ICG	0.1–0.3%	780	1000–1500	Clinical fluorescence angiography, NIR-II microsurgery	[18,19,20,21]
BBTD	1–2%	540–730	1000–1200	Fluorescence and photoacoustic angiography, tumor imaging	[22,23,24,25,26]
BIBDAH	**	1028	1000–1700	Fluorescence angiography in mouse model, photoacoustic imaging	[27,28]
SPN	0.1–50%	980	1000–1300	Fluorescence tumor imaging in mouse model, bone marrow imaging	[29,30,31,32]
Porphyrin	0.1–0.5%	860	1000–1200	Fluorescence tumor imaging in mouse model, PAI in mouse model	[33,34,35]
AIE	0.05–48.6%	808–895	1000–1600	Fluorescence angiography, lymphatic and tumor imaging, PAI in mouse model	[36,37,38,39,40]

** Not known. ICG = Indocyanine green; BBTD = benzobisthiadiazole; BIBDAH = butyl diphenylaminocyclopentene; SPN = semi-conducting polymer nanoparticle; AIE = aggregation-induced emitter.

**Table 2:** Photophysical properties and applications of major inorganic NIR-II fluorophores.

Inorganic Fluorophore	Quantum Yield	Peak Excitation Wavelength (nm)	NIR-II Emission Wavelength (nm)	Bioimaging Application	Reference
AgS QD	20–40%	350–450	1000–1400	Fluorescence tumor imaging in rat and mouse models	[41,42,43,44,45]
PbS QD	40–60%	400–800	1000–1700	Infection monitoring, fluorescence angiography in mouse model, tumor imaging, monitoring of collagen degradation in mouse	[41,46,47,48,49]
SWCNT	1–10%	300–800	1000–1700	Dual modal fluorescence and PAI, cerebrovascular and tumor imaging	[41,44,50,51]
RENP	20–80%	300–800	1000–1600	Fluorescence tumor imaging in rat model, organ imaging	[44,52,53,54,55]
Nanogolds	1–10%	400–800	1000–1100	Fluorescence tumor imaging in rat model, imaging of bones	[56,57,58,59]
Graphene materials	0.02–70.3%	UV–NIR	**	PAI of tumor tissue in animal models	[60,61,62]
Carbon dots	0.04–1.27%	UV–VIS	1000–1200	Fluorescence gastric imaging and pH sensing	[63,64,65,66]

** Not known; QD = quantum dot; SWCNT = single-walled carbon nanotube; RENP = rare-earth nanoparticle.

## 3. Major Classes of NIR-II Fluorescence and Photoacoustic Fluorophores

This section discusses organic and inorganic fluorophores for fluorescence and photoacoustic bioimaging in the NIR-II window. It begins by highlighting the features of organic fluorophores and their limitations, such as low photostability and the lack of biocompatible dyes emitting wavelengths exceeding 1200 nm. It then delves into various classes of organic fluorophores, such as cyanine dyes, organic electron donors and acceptors, commercial donor–acceptor IR dyes, aggregation-induced emitters, and semiconducting polymer nanoparticles. For each class, specific examples, applications, and challenges are discussed. The section then transitions to inorganic fluorophores, focusing on semiconductor quantum dots, graphene-based materials, single-walled carbon nanotubes, rare-earth nanoparticles, nanogolds and gold nanomaterials, and carbon dots. Throughout the discussion, attention is paid to their unique properties, applications, and prospects in NIR-II fluorescence and photoacoustic imaging. 

### 3.1. Organic Fluorophores

**Figure 2:**
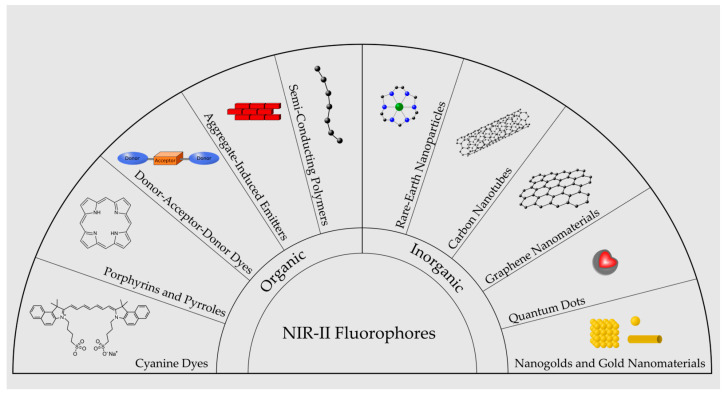
Illustration of major categories of NIR-II emitting fluorophores.

Organic dyes are the first class of contrast agents developed for fluorescence bioimaging. Organic dyes generally have low photostability but have low cost and well-established functionalization chemistry and safety assessments. There is also a lack of organic, biocompatible dyes that emit wavelengths exceeding 1200 nm [67]. Major classes of NIR-II fluorophores are illustrated in Figure 2.

#### 3.1.1. Cyanine Dyes

Cyanide dyes represent a sub-type of organic small-molecule fluorescence probes. NIR fluorophore design is commonly based on the modification of polymethine dyes. The most well-known cyanine dye, indocyanine green (ICG), is an FDA-approved imaging contrast used for angiography in ophthalmology. ICG has a half-life of 2–4 min in aqueous solution and binds rapidly to serum proteins, resulting in fast clearance [68]. ICG also has a recorded image depth of 3 mm and molar extinction coefficient of 170,000 M^−1^cm^−1^ [69,70].

Although ICG exhibits peak fluorescence emission at 820 nm, it is known to exhibit long-tail fluorescence in the SWIR wavelengths of up to 1500 nm [18]. This property allows ICG to serve as a benchmark for experimental SWIR fluorescence probes [16]. Analogues of ICG have been synthesized to shift the emission maxima. Additionally, various packaging and encapsulation techniques are being studied to increase photochemical stability and targeting accuracy. 

ICG has also been studied as a contrast agent in PA cancer imaging [71]. Lee et al. have characterized the PA signal characteristics of ICG agar phantoms. Their study found ICG PA intensity to increase logarithmically without quenching effect for concentrations up to 5 mg/mL. PA signal intensity was observed in tissue depths of 22 mm.

#### 3.1.2. Organic Electron Donors and Acceptors

Benzobisthiadiazole (BBTD, BBT) is an organic electron acceptor applied to NIR-II functional imaging with a 3 mm image depth [22,72]. BBTD contains four electron deficient C=N bonds, making it an attractive choice for D–A-based conjugated polymers [72]. The bandgap for such engineered polymers must be less than 1.24 eV. Tuning the bandgap of D–A polymers relies on the selection of D and A units to produce the intramolecular charge transfer (ICT) effect. The bandgap is formed by the hybridization of molecular orbitals post-polymerization to produce a new higher-lying highest occupied molecular orbital (HOMO) and a newer low-lying lowest unoccupied molecular orbital (LUMO). Recently, BBT has been modified to improve solubility in biological systems. Replacing the thiadiazole ring of BBT with a triazole or pyrazine ring produces thiadiazolobenzotriazole (TBZ) or thiadiazoloquinoxaline (ATQ, TTQ). These BBT derivatives introduce alkyl side chains to improve polymer solubility. 

Coupled with a strong electron donor, the strong donor–strong acceptor strategy has yielded NIR-II-sensitive polymers based on the ICT effect. Typical electron donors coupled with BBT or thiophene-based acceptors include cyclopentadithiophene (CDT), dithienosilole (DTS), and dithienopyrrole (DTP) [73].

#### 3.1.3. Commercial Donor–Acceptor IR Dyes

Several polymethine molecules such as IR-26, IR-783, IR-1048, IR-1051, and IR-1061 exhibit fluorescence in the NIR-II region [74]. Many, like ICG, were designed to have a short-wave peak emission and were later discovered to have a long-tail emission into the NIR-II. IRDye800CW is being tested in the clinical setting. 

Kilian et al. obtained and screened 13 commercially available dyes with NIR-II absorbance for in vivo fluorescence and PA imaging [27]. Of these, the cyanide dye benzo indole butyl diphenylaminocyclopentene heptamethine tetrafluoroborate (BIBDAH) has stood out for its high absorbance at 1064 nm and its stability in formulations of biocompatible surfactants. BIBDAH exhibits a peak emission wavelength at 1070 nm when excited with 980 nm light and produces a long emission tail up to 1300 nm. As a PA contrast, BIBDAH was observed at a 2.5 mm depth in a tissue-mimicking phantom and in pancreatic subcutaneous tumors after intravenous injection in nude mice. The dye was next assessed as a blood vessel contrast in mice. Observed blood vessels showed at least a 25% greater signal than background from adjacent tissue. 

Kilian et al. continued to investigate BIBDAH as a NIR-II PA contrast agent for longitudinal pre-clinical imaging [28]. Here, BIBDAH was formulated with the surfactant HS15 Kolliphor, which resulted in a peak absorbance of 1036 nm. PA signal intensity was shown to increase linearly as a function of concentration in an intralipid phantom. In vivo imaging of mice administered with BIBDAH via an intraperitoneal route showed a consistent contrast signal intensity over two days. The organic dye LZ-1105 was developed by Li et al. as a small molecule NIR-II probe for vascular imaging [75]. LZ-1105 demonstrated a higher fluorescence intensity than ICG when compared at their respective peak excitation frequencies.

#### 3.1.4. Semiconducting Polymer Nanoparticles

Semiconducting polymer nanoparticles (SPNs) are a subclass of organic semiconducting materials composed of highly π-conjugated, electron-delocalized polymer backbones, which may be modified for bioimaging [41]. Like quantum dots (QDs), SPNs have large absorption coefficients, high photostability, a tunable size, and optical parameters [76]. However, a higher biocompatibility distinguishes SPNs from QDs. SPNs exhibit a large range of quantum yield due to factors inherent to their structure, including size and morphology and chemical composition, as well as external factors such as solvent polarity and temperature. SPNs may be adapted for NIR-II fluorescence or PA imaging [41]. 

Relatively few SPNs have been engineered toward NIR-II fluorescence imaging as small-molecule fluorophores in NIR-II fluorescence imaging are limited by low fluorescence quantum yields. However, Hong et al. synthesized a polymer fluorophore to emit in the NIR-II window—the first to do so [29]. The polymer, pegylated and composed of poly (benzo [1,2-b: 3,4-b0] difuran-alt-fluorothieno-[3,4-b] thiophene) (named ‘pDA’), has a fluorescence quantum yield of 1.7% and is tuned to an emission peak of 1040 nm. This enabled the real-time tracking of arterial blood flow in the mouse at 25 frames per second in the NIR-II window. In a separate study, an advanced imaging technique called optical resolution photoacoustic microscopy (ORPAMI) was employed along with bright near-infrared II (NIR-II)-conjugated polymer nanoparticles (CP NPs) to accurately identify tumor and cerebral tissue vasculatures [77].

The generally high photothermal conversion efficiencies of SPNs are advantageous for PA imaging. Zhen et al. outlined a screening process to elucidate the relationship between SP molecular structure and PA properties [76]. They demonstrated that PA signals from SPNs are closely dependent on nonradiative thermal deactivation pathways and showed a negative relationship between PA signal intensity and fluorescence intensity for SPNs of varying compositions. This further demonstrates the direct competition between the nonradiative thermal deactivation pathway with the fluorescence emission pathway.

#### 3.1.5. Aggregation-Induced Emitters

Many organic fluorophores exhibit aggregation-induced quenching, which may preclude their use as solid-state nanoparticles. On the other hand, some fluorophores become brighter in the solid state, a phenomenon called aggregation-induced emission (AIE) [78]. Fluorophore-doped nanostructures take advantage of this property by encapsulating AIE lumogens within polymer matrices. Such polymers have enhanced water dispersibility, biostability, and tissue targeting capability. AIE-based NPs mostly emit in the visible and NIR-I ranges; designing NIR-II-sensitive AIEgens remains a challenge. 

Wu et al. designed a novel AIEgen with emission in the NIR-II region based on donor–acceptor–donor (D–A–D)-structured small molecules [36]. The absorption and emission profiles of D–A–D-structured small molecules rely on the chemical properties of the constituent molecules, rather than the size of the fluorophore. Their AIE NPs, dubbed “L897”, exhibited an emission tail extending to 1200 nm and a fluorescence quantum yield of 5.8%. The group performed in vivo blood vessel imaging, lymphatic imaging, and tumor imaging with NIR-II fluorescence. Li et al. synthesized AIEgens capable of NIR-IIa and NIR-IIb fluorescence imaging [37]. Guo et al. reported the first dual-functioning NIR-II fluorescence and PAI NP polymer [38]. Following the AIE molecular design principle, Liu et al. developed highly efficient NIR-II-emitting and AIE nanoprobes (2TT-oC6B) using BBTD as the acceptor, thiophene as the bridge and donor, and triphenylamine as the second donor. The resulting 2TT-oC6B probe displayed a prominent emission peak at 1030 nm and achieved a high quantum yield of 11% [39].

Singh et al. reported J-aggregates (JA) made of indocyanine green dye as a candidate for a biocompatible NIR contrast agent class suitable for photoacoustic imaging [40]. They introduced a synthesis method to create ICG aggregates with adjustable sizes as small as 230 nm. The developed ICG-JA platform exhibits a notable in vitro photoacoustic (PA) signal, which is twice as strong as whole blood, along with remarkable photostability. Near-infrared photoacoustic imaging (NIR-PAI) in the mouse model demonstrated an improved visualization of the liver and spleen for up to 90 min post-injection, achieving a contrast-to-noise ratio of 2.42.

#### 3.1.6. Porphyrin- and Pyrrole-Based Polymers

Porphyrins are large, heterocyclic compounds consisting of four subunits of pyrrole linked by methane bridges, which may be substituted by functional groups at the β-position [79]. Porphyrins and porphyrin derivatives represent a diverse array of compounds ubiquitous in biological systems, including heme and chlorophyll. Free-base porphyrins may incorporate a metal ion in the center of the porphyrin to form a metalloporphyrin. Porphyrins generally have high photochemical stability due to their large π-aromatic system, but poor water solubility. Typical porphyrins have the strongest absorption in the visible light spectrum, although molecular engineering techniques are producing NIR-II-sensitive compounds. 

Boron-dipyrromethene (BODIPY)-based compounds are a class of NIR-II-emitting pyrroles [80]. Zou et al. developed three NIR-II-sensitive BODIPY derivatives for photothermal therapy applications [81]. However, NIR-II imaging was not performed. Bai et al. engineered a class of BODIPY dyes that exhibit bright NIR-II fluorescence and photostability, although with low water solubility [82]. Shi et al. introduced a fluorescent BODIPY nanoplatform (InTBOD-Cl) to enable NIR-II emission during drug release for targeted cancer imaging [83].

### 3.2. Inorganic Fluorophores

#### 3.2.1. Semiconductor Quantum Dots

Quantum dots (QDs) are inorganic semiconductor nanocrystals with narrow, tunable fluorescence emission bands and broad absorption bands. QDs are bright, highly photostable, and easily conjugated, making them desirable for fluorescence bioimaging. QDs have potential for heavy metal toxicity and for relatively short circulation time [84].

Lead sulfide (PbS) QDs have the highest fluorescence quantum yield (60%) and can be tuned to emit in the NIR-II wavelengths. However, they have a relatively low chemical stability and have been criticized for potential toxicity from the leaking of heavy metal ions resulting from sulfide oxidation. Zebibula et al. addressed the issue of toxicity by encapsulating PbS QDs in dual layers of Pluronic F-127 polymer [85]. Zhang et al. coated PbS QDs with a layer of cadmium sulfide (CdS) to protect against the oxidation of the PbS core [86]. This resulted in a higher biocompatibility in the mouse model, but blue-shifted the emission peak from 1725 nm to 1650 nm. Imamura et al. demonstrated that a concentration of 3000 nM of PbS QDs in mice did not cause acute toxicity and presented a clear NIR fluorescence image of cerebral blood vessels [46]. In a study by Kong et al., PbS nanocrystals capped with protein molecules prevented the release of lead content, which was validated in the mouse model by comparing neurite connections between a control group and a group treated with a high dose (~1 μM) of QDs [87]. These QDs displayed a Gaussian emission peak at 1308 nm, which allows for the imaging of tissue structures at a depth of 1.5 cm.

PbS/CdS nanoparticles have a limited water solubility due to a coating of oleylamine during synthesis, which poses a challenge for deployment in living systems. Jin et al. developed several PbS QDs to overcome water solubility issues, including coating the QDs with glutathione (GSH) and recombinant proteins [88]. Zhao et al. enhanced the water solubility of PbS QDs through capping three different amphiphilic ligands (OLA, OA, OA/TOP) [89]. However, all three ligands produced blue-shifted emission spectra and an approximate 60% decrease in emission intensity. Tian et al. synthesized a dual NIR-II probe pair, namely the donor–acceptor–donor (D–A–D) fluorescent probe IR-FD and PbS quantum dots (QDs), for NIR-II imaging of the resection of metastatic proximal lymph nodes. The synthesized D–A–D fluorescent probe IR-FD facilitated fluorescence imaging of primary/metastatic cancer within the NIR-IIa spectrum (1100–1300 nm), while PbS QDs were employed to detect cancer-invaded sentinel lymph nodes in the NIR-IIb (>1500 nm) window [90]. Yang et al. synthesized biocompatible PbS QDs to monitor the migration of injected mesenchymal stem cells (MSCs), as well as gather information about their biological distribution and clearance. They further applied these QDs in the treatment of supraspinatus tendon tears in mice [91].

Silver sulfide (AgS) QDs are among the most successful for live cell tracking due to high fluorescence quantum yield in the NIR-II window [84]. Although quantum yields for QDs are generally very high (20% or greater), most absorbed light energy is converted to heat through several nonradiative relaxation pathways [92]. Ren et al. developed a synthesis process for AgS QDs functionalized with carboxyl and amino groups, which demonstrated an increased aqueous stability [42]. Conditions of the synthesis process are controlled to tune the emission spectra by modifying the size of the particles. This group imaged HeLa cells tagged with NIR-II-emitting QDs. By employing ligand exchange, AgS QDs can be modified to enhance their hydrophilicity by introducing defects into the nanocrystal structure. While the hydrophilized QDs exhibit a decrease in fluorescent yield, they also demonstrate a red-shifted emission spectra in comparison to their unmodified counterparts. Rotko et al. developed hydrophilized QDs that were stable in aqueous dispersions for 12 months without the loss of luminescence [93]. Huang et al. developed AgS quantum dots (QDs) for the purpose of visualizing the distribution of transplanted hMSCs within the body [94]. Shashkov et al. demonstrated PA signals acquired from core-shell CdSe/ZnS Qds. PbS nanocrystals, however, which demonstrate very weak PA signals, are generally not used for photoacoustic imaging [95].

#### 3.2.2. Graphene-Based Materials

Graphene is one of the most well-studied nanomaterials, consisting of a two-dimensional sheet of sp^2^ hybrid bound carbon atoms in a hexagonal configuration [96]. Graphene and graphene-based derivatives such as graphene oxide and reduced graphene oxide represent a diverse array of sheet-shaped NIR-II contrast agents. These sheets may be stacked together to form stacks. Graphene oxide (GO), an oxidized form of graphene, may be engineered with hydrophilic moieties such as hydroxyl and carboxylic groups to improve aqueous solubility. GO may be engineered to have a varying ratio of carbon to oxygen atoms, but too much oxygen makes the structure unstable; oxidation breaks the sp^2^-hybridized structure, generating wrinkles in the sheet. Reduced graphene oxide (rGO) features further functional defects across the basal plane to enable the loading of various other materials [97]. However, the reduction process decreases the water solubility of rGOs. 

Reduced GO shows strong fluorescence quenching when absorbing NIR light; however, NIR-II emission has not been studied. Of the graphene-based contrasts, rGOs are especially suited toward PAI because their larger sp^2^ domains more efficiently absorb NIR light [60]. This property may be enhanced with the loading of additional NIR-II light-absorbing substances. Wang et al. developed a sandwich-type rGO-AuNP nanocomposite for PAI in the NIR-II window [61]. The rGO-AuNP produced a higher contrast from NIR-II PA excitation than NIR-I PA excitation after injection into tumor-bearing mice. A graphene quantum dot with NIR-II absorbance properties was developed by Liu et al. [62].

#### 3.2.3. Single-Walled Carbon Nanotubes

Single-walled carbon nanotubes (SWCNTs) are a family of highly elongated tubular structures composed of covalently bonded carbon atoms arranged in ordered forms. Carbon nanotubes are a high-aspect ratio nanostructure of sp^2^-hybridized carbon atoms, constructed from graphene sheets rolled into tubes at a chiral angle [98]. The resulting structure is a quasi-one-dimensional wire with a diameter of 0.4–2 nm and a length to up to 1 cm. SWCNTs are a distinct graphene-based contrast for NIR-II imaging for their unique optical properties. 

The optical properties of SWCNTs offer unique advantages, including high photostability under a prolonged NIR fluorescence. This non-photobleaching property of SWCNTs lends advantage for long-term measurements in real-time. SWCNTs have a relatively low fluorescence quantum yield due in part to quenching by structural defects [50]. Fluorescence can be selectively modulated through the adsorption of redox-active molecules to the surface of the SWCNT. The resulting electron transfer affects the fluorescence spectrum of the SWCNT via the bleaching or quenching of excitons. In addition, the adsorption process changes the dielectric environment around the SWCNT by replacing solvent molecules, causing a solvatochromatic shift in the fluorescence. 

Hong et al. developed a dual-color SWCNT to characterize differences between NIR-I and NIR-II fluorescence imaging [99]. Their NIR-II-absorbing SWCNTs were functionalized with IRDye-800, a commercially available NIR-I fluorophore for imaging blood vessels in the mouse. The study demonstrated a superior spatial resolution of NIR-II fluorescence over NIR-I. The same group proceeded to use this same contrast to resolve blood capillary vessels in the mouse brain at a millimeter depth [100]. In this study, the NIR-IIa window demonstrated a lower tissue scattering than NIR-I wavelengths. In a study by Diao et al., SWCNT NIR-IIb emitters resolved cerebral mouse vasculature with a higher signal-to-background ratio than NIR-I and NIR-II imaging [2]. 

Photoacoustic signal has been recorded from SWCNTs of lengths 50–300 nm and diameters of 1–2 nm [101]. Photoacoustic signal increased linearly as a function of molar concentration when excited at 1064 nm wavelength. Although the SWCNTs had been conjugated with RGD for imaging, the RGD was shown to have no statistical effect on optical absorbance or photoacoustic signal. Another group demonstrated an enhanced photoacoustic signal of SWCNTs conjugated with ICG [102].

#### 3.2.4. Rare-Earth Nanoparticles

Rare-earth (RE) nanoparticles are composed of biocompatible lanthanide metals and feature high photostability and narrow-band fluorescence emission [103]. In terms of size, RE nanoparticles are classified as either inorganic nanocrystals or organic ligand complexes. In terms of luminescence mechanism, RE nanoparticles can further be divided into two categories: up-converting and down-converting. Photon up-conversion occurs when the emission wavelength is shorter than the excitation wavelength, while down-conversion results in an emission wavelength longer than the excitation wavelength. Several competing processes can interfere with the down-conversion emission once the RE ion has been excited to its resonance state. Up-conversion to visible or ultraviolet emission, and non-radiative cross-relaxation between neighboring RE ions can each interfere with down-conversion fluorescence. Additionally, there is a strong energy transfer rate between excited RE ions and OH^−^ ions, resulting in aqueous quenching. Up-conversion luminescence has been well studied for decades in the context of imaging. Down-converting RENPs are less studied yet hold greater potential for NIR-II imaging.

Erbium-based nanoparticles (ErNPs) have been studied as promising NIR-IIb fluorescence contrast agents. The excitation of the Er^3+^ ion with 980 nm light produces the resonance energy required for a down-conversion fluorescence emission at 1550 nm. Erbium lattices may be structured in a compact, hexagonal configuration (β-phase) or cubic configuration (α-phase). These structural phases have been shown to influence NIR-II fluorescence of ErNPs. Zhong et al. reported that zinc-doped alpha-phase ErNPs produced an eleven-fold enhancement of down-conversion fluorescence [104]. 

The RE ion may be excited directly via absorption, or receive energy from a neighboring ion, a process known as sensitization. Ytterbium ions have been shown to be sensitizing agents in nanoparticle formulations for down-conversion luminescence, first demonstrated by Naczynski et al. [52]. The resonant transfer of energy from the Yb sensitizer to the Er activator results in the emission of the ErNP at 1550 nm. 

Few studies have investigated the NIR-II excitation of PA RENPs. However, He et al. increased the PA efficiency of up-converting RENPs by functionalization with acrylic acid [105]. This platform enabled dual modal NIR-II fluorescence imaging and PAI in the mouse model. Ren et al. presented their findings on a highly dispersed nanoprobe doped with Nd^3+^ ions and featuring a core-shell structure (NaYF_4_:5%Nd@NaGdF_4_). This nanoprobe exhibited a long-tailed emission spectrum in the NIR-II range. Surface modification of the nanocrystals using DSPE-PEG_2k_ extended blood circulation time and enhanced biocompatibility and biosafety when tested in vivo. The nanoprobe exhibited a relatively high quantum yield of 0.52%, surpassing that of the fluorescence dye IR-26. Utilizing NaYF_4_:5%Nd@NaGdF_4_ as the basis for NIR-II imaging, the successful visualization and monitoring of tumor blood vessels were achieved in breast tumor models [106].

#### 3.2.5. Nanogolds and Gold Nanomaterials

Nanogolds represent a family of nanostructures (nanorod, nanosphere, nanocube, etc.) studied for potential in cancer theranostics and NIR-II imaging. Gold nanomaterials are biocompatible, chemically inert, and typically have high molar extinction coefficients. Nanogolds in the size range of 5–200 nm are captured by the reticuloendothelial system, which may place internal organs at risk. However, atomic gold nanoclusters (AuNCs) have sizes below the kidney filtration threshold, which allow for rapid renal clearance.

Kong et al. developed AuNCs tailored for NIR-II fluorescence imaging in a xenograft-bearing mouse model [56]. Such AuNCs exhibit a quantum yield of 0.21% and a gaussian emission profile centered at 1050 nm, with a full width half maximum of 200 nm. AuNCs synthesized by Baghdasaryan et al. produced similar quantum yields and emission profiles [107]. No cytotoxicity was observed in murine cancer cells incubated with varying concentrations of AuNCs. In both cases, the AuNCs were easily conjugated with cancer cell-targeting moieties. 

Gold nanomaterials have been studied as an NIR-II-absorbing PA contrast. Of these, miniature gold nanorods (solid or hollow) that are 5–10 times smaller than regular nanorods have emerged as an attractive choice due to high structural aspect ratios, as plasmonic absorption is closely related to particle size and structure. Cai et al. developed such a miniature hollow gold nanorod (~46 nm) for cancer-targeted PAI in the mouse model [107]. Functionalizing this gold nanorod probe with porphyrins granted fluorescence contrast capability. The Chen group produced small (~49 nm) gold nanorods, which demonstrated superior PA intensity and photostability in comparison to larger (~120 nm) gold nanorods [108].

#### 3.2.6. Carbon Dots and Graphene Dots

Carbon dots (CDs) are small carbon nanoparticles typically ranging from 2 to 10 nanometers in size. They are derived from carbon-rich precursors through a simple and low-cost synthesis process, often involving hydrothermal or microwave-assisted methods [109]. The exact structure and properties of carbon dots can vary depending on the synthesis conditions and precursor materials used. Graphene dots, also known as graphene quantum dots (GQDs), are a subclass of carbon dots that are derived from graphene. These are typically much smaller in size than the graphene materials previously mentioned, ranging from 1 to 10 nanometers. Carbon and graphene dots hold several key advantages, including low-cost, low-toxicity, low-environmental impact, strong fluorescence, and high thermostability and photostability [110]. However, carbon dots mostly absorb in the UV–VIS window, which is not ideal for bioimaging. Carbon dots have been engineered for photoacoustic imaging using visible and NIR light excitation; however, none have been designed to absorb NIR-II wavelengths [111]. 

Carbon dots are primarily characterized by their intrinsic fluorescence, with the latest property under investigation being NIR-II fluorescence. Carbon dots doped with iron (Fe-CDs) developed by Ci et al. exhibited a linear, pH-dependent fluorescence intensity peaking at 1000 nm [63]. These were successfully used to sense gastric acidity in the mouse model and displayed low cytotoxicity in cellular assays. Few graphene dots have been synthesized to exhibit NIR-II fluorescence; however, Wang et al. developed GQDs for the NIR-II imaging of internal organs and blood vessels in the mouse [65]. 

## 4. Delivery Technology

**Figure 3:**
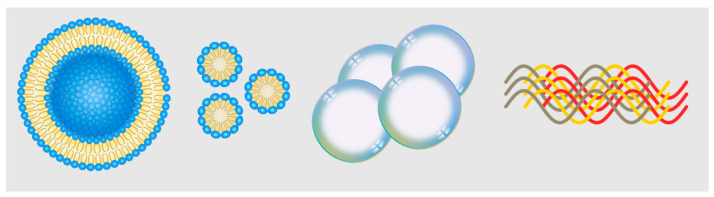
Representative schematics for nanoscale delivery technologies. Left to right: liposome, nanomicelle, nanobubbles, protein complex.

The main contents discussed in this section focus on different types of delivery systems for imaging contrast agents. The first part discusses lipid-based delivery systems, which offer formulation versatility, biocompatibility, high payload content, and stability for NIR-II contrast agents. The second part introduces microbubble-based delivery, which involves microspheres of encapsulated gas that can be used as ultrasound contrast agents and drug delivery platforms. NIR-II fluorescence imaging using microbubbles has been explored, showing potential for multi-modal imaging applications. Next, nanomicelle-based delivery systems are discussed, focusing on polymerized micelles. Lastly, protein assemblies for imaging are presented, involving supramolecular complexes of proteins loaded with fluorophores. These various delivery systems, illustrated in Figure 3, provide valuable tools for advanced imaging applications.

### 4.1. Lipid-Based Delivery

Lipid-based carrier systems are an emerging technology designed to address issues with biocompatibility and water solubility in delivered biomolecules [112]. Lipid-based delivery systems represent an entire family of varying structural and chemical formulations, including lipid nanoparticles, liposomes, micelles, emulsions, and lipid carriers. The main properties affecting the choice of lipid-based formulations are solubility, dispersion, digestion, and absorption. Pouton’s classification formally classifies lipid-based delivery systems based on the triglyceride content, surfactant content, hydrophilic cosolvent content, particle size of dispersion, significance of aqueous dilution, and significance of digestion. Lipid-based delivery systems have the advantages of formulation versatility, biocompatibility, high payload content, and pharmaceutical stability. 

Lipid encapsulation techniques are being applied to NIR-II contrast agents. In a study, IR-1061 dye was encapsulated within a lipid nanoparticle functionalized with PEG for the PA imaging of hepatocellular carcinoma in mice [113]. This Polipo-IR NP demonstrated PA sensitivity with a concentration as low as 1.03 ug/mL and laser energy as low as 1 mJ. Photostability was established after exposure to 4000 consecutive laser pulses. In mice-bearing HCC, the Polipo-IR NPs were internalized by Hep-G2 cells. No significant tissue denaturation or organ damage, nor abnormal changes to body weight or daily behavior was observed in mice during 18 consecutive days after treatment, suggesting no obvious toxicity.

### 4.2. Microbubble-Based Delivery

Microbubbles are microspheres of encapsulated gas commonly used as ultrasound contrast agents but have gained recent attention as a platform technology for drug delivery and multi-modal imaging [114,115]. The microbubble shell may be composed of lipids, proteins, saccharides, or polymers, and may be functionalized via covalent attachment and even designed to be destroyed under selective sound wavelengths. Ultrasound waves have the advantage of creating transient, non-lethal perforation in cell membranes, a process known as sonoporation. The presence of microbubbles greatly decreases the amount of energy needed for cavitation to occur. Microbubbles doped with ICG designed by Liang et al. have traversed the blood–brain barrier in this way for NIR-II fluorescence imaging [116]. Recently, porphyrin shell lipid microbubbles have demonstrated NIR fluorescence in vitro [117]. Wang et al. synthesized nanobubbles consisted of lipid shells with a C_3_F_8_ gas core loaded with Sudan Black (BNB) and DiD fluorescent dye. This was applied as a photoacoustic contrast agent for breast cancer imaging and showed the potential of BNBs as multi-modal contrast agents capable of specialized tumor imaging in vivo [118].

### 4.3. Nanomicelle-Based Delivery

Polymerized micelles, also known as nanocarriers, have potential for delivering imaging contrast agents. These nanomicelles are self-assembled, colloidal dispersions with a hydrophobic core and hydrophilic shell, typically 10–100 nm in diameter [119]. Micelles are structurally stable, biodegradable, and are particularly advantageous for carrying payloads with low solubility in water. Lactosomes, a well-studied class of nanomicelle, are composed of polymerized poly-L-lactic acid and poly-sarcosine. Lactosomes loaded with ICG demonstrated NIR fluorescence in vivo [120]. Lactosome conjugation with PEG and peptide-targeting moieties has been performed in previous studies [121].

### 4.4. Protein Assemblies

Supramolecular assemblies of protein complexes may be loaded with fluorophores for labeled imaging. These bioengineered platforms can be tuned to enhance the brightness of the fluorophore in addition to modifying the pharmacokinetic and clearance properties of the probe. Antaris et al. functionalized the NIR-II dye CH-4T to form bright supramolecular assemblies with plasma proteins [122]. Such protein complexes were demonstrated to enhance the fluorescence intensity of CH-4T when mixed in serum compared to fluorophores suspended in saline. This effect is hypothesized to result from the binding of the dye to the protein; there is a reduced aggregation-induced self-quenching of the fluorophore in addition to an enhanced geometrical confinement, resulting in a higher quantum yield. The Tian group has developed a strategy for designing proteins to uptake NIR-II-sensitive dyes [123]. An albumin chaperone protein acted as a brightness amplifier for cyanine-based dyes. This enhanced brightness is hypothesized to originate from covalent binding interactions between the cyanine dye and albumin protein. Of note is the IR820-HSA probe designed by Du et al., which demonstrated dual NIR-II fluorescence and PAI capabilities [124]. In each of these cases, the quantum yields of the respective fluorophores were enhanced after assembly within a protein complex.

## 5. Functionalization and Surface Treatment

**Figure 4:**
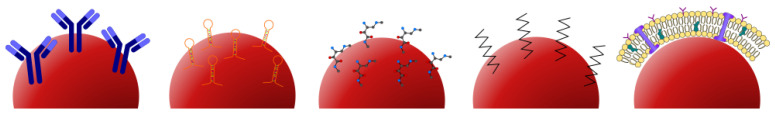
Left to right: representative fluorophores conjugated with antibodies, aptamers, peptides, polyethylene glycol (PEG), and cell membrane encapsulation.

This section focuses on the functionalization and surface treatment of imaging contrast agents. PEGylation is explored as a biochemical modification process involving the attachment of polyethylene glycol (PEG), which allows for the retention of imaging contrast agents without the loss of function. The second part discusses the use of monoclonal antibodies and antibody mimetics for contrast-enhanced imaging. The third part covers targeting peptides, which are small peptide sequences capable of binding to cells and tissue targets with high specificity and affinity. Aptamers, which are synthetic RNA or single-stranded DNA molecules, represent an emerging class of ligands for contrast agent delivery. Lastly, cell membrane encapsulation is discussed. Each of these are illustrated in Figure 4. This section presents various strategies for functionalizing and surface-treating imaging contrast agents, enhancing their performance for specific targeting and improved imaging capabilities. 

### 5.1. PEGylation

PEGylation is a biochemical modification process of covalent or non-covalent attachment of polyethylene glycol (PEG) to biomolecules [125]. Composed of repeating units of ethylene glycol, PEG has been shown to enhance water solubility, half-life, and reduce the immunogenic response of modified proteins. PEG molecules may be branched or linear, which alters the physiochemical properties of the conjugated biomolecule. PEGylation leads to the retention of imaging contrast agents without the loss of function. 

PEG is synthesized from ethylene oxide monomers by means of ring-opening polymerization. The reaction produces a mixture of bi-functional and mono-functional PEG chains of varying molecular weights. The size and position of PEG molecules strongly affect the properties of the conjugated biomolecule. First-generation PEGylation relied on the non-specific binding of PEG chains of varied sizes, but additional purification steps are employed in recent methods to improve the specificity of function.

### 5.2. Monocolonal Antibodies and Antibody Mimetics

Monocolonal antibodies (mAbs) are highly site-specific proteins developed for biotherapeutics [126]. The affinity properties of mAbs allow for precise binding in contrast-enhanced imaging. The key challenges to mAbs are that they are costly to manufacture and are unstable during transport and storage. Antibodies conjugated with ICG for NIR-II fluorescence imaging in tumor-bearing mice were reported by Shi et al. [127]. It was found that each GPC3 antibody probe was labeled with two ICG molecules. The GPC-ICG probe provided a tumor to background ratio of 3, higher than ICG alone. Water-soluble SWCNTs functionalized with PEG and integrin antibodies for PA molecular imaging in vivo were reported by Xiang et al. [128]. These leveraged the NIR-absorbing properties of SWCNTs for early tumor detection in mice. Biocompatibility was verified with in vivo systemic toxicity tests over the course of two months.

### 5.3. Targeting Peptides

Cell-targeting peptides (CTPs) are small peptide sequences capable of binding to cells and tissue targets with high affinity and specificity [129]. CTPs represent a growing research area as a new class of ligands capable of delivering therapeutic and diagnostic agents. Peptide sequences (3 to 10) are easy to manufacture, have small size, low immunogenicity and cytotoxicity and provide comparable binding properties to antibodies. Additionally, peptides may be engineered with controlled cargo-release mechanisms in response to physical or chemical stimuli [130]. Recent reviews detail peptide sequences evidenced to target specific tissues or cell types [131]. Several strategies exist for identifying peptide sequences and their associated cell targets: the study of structure–activity relationships, phage display library, and chemical-library-generation techniques. Such techniques have been applied toward the delivery of fluorescence and PA contrast agents. 

Several peptide-conjugated NIR-II fluorophores have been reported. Wen et al. applied an amphiphilic peptide to AgS quantum dots conjugated with the tumor targeting RGD peptide [43]. This chain-like nanoprobe assembly exhibited capability for cancer cell detection in a rat model.

### 5.4. Oligonucleotide Aptamers

Aptamers are synthetic sequences of RNA or single-stranded DNA molecules that represent an emerging class of ligands for contrast agent delivery. The SELEX (systematic evolution of ligands by exponential enrichment) method is used to generate a nucleic acid sequence from a combinatorial library [132]. Compared to antibodies, aptamers are easy and inexpensive to synthesize. High clearance rates in vivo can be prolonged via conjugation with PEG or cholesterol. Aptamer targets include cell surface proteins and small molecules. 

An analysis by Kelly et al. compared the binding properties of fifteen aptamers to eleven different cancer lines as well as to that of antibody controls [133]. This study established standard methods of identifying and evaluating aptamers designed for cell surface targeting. Aptamers were shown to exhibit some non-specific binding to proteins and other cellular components due to their innate negative charge. Different cell types demonstrated varying rates of non-specific background binding. Although aptamers may have high binding affinity comparable to that of antibodies, this does not indicate how robustly it will function in cell or animal models. 

However, aptamers have been successfully conjugated on fluorescence and PAI probes. An in vivo study by Shi et al. demonstrated a high specificity of tumor cell binding using a cyanide-based dye labeled with the TD05 aptamer [134]. PA probes based on aptamers have been developed using the NIR fluorophore/quencher pair IRDye 800CW/IRDye QC-1 [135]. Such aptamers are notable for forming a complex when the two moieties are in proximity. The activation of the probe cleaves this complex and eliminates contact quenching. The adaptable properties of aptamers lend to potential conjugation with NIR-II-sensitive substances.

### 5.5. Biomimetic Encapsulation

Biomimetic functionalization is an emerging approach of NIR-II nanoprobe surface treatment involving the encapsulation of fluorophores within cell membrane components. Often referred to as cell membrane-coated nanoparticles (CCNPs) or camouflaged nanoparticles, biomimetic materials are encapsulated within sections of an extracted cell membrane, granting them native cell functionalities. This membrane can be further treated with additional surface ligands and modifications. Biomimetic encapsulation may be considered both a delivery system and a surface treatment, as the cell membrane imparts properties of both functionalities to the nanoprobe. 

Biomimetic encapsulation is particularly advantageous for the delivery of nanoprobes across the blood–brain barrier (BBB). Men et al. recently demonstrated specific NIR-II fluorescence imaging of glioma in a mouse model using membrane-coated polymer dots [136]. Similarly, Huang et al. encapsulated metallic quantum dots within neural stem cell membranes for trans-BBB delivery in the mouse model [137]. NIR-II fluorescence imaging verified their presence in mouse brain. In each of these cases, the nanoprobes were up-taken and trafficked to the brain by epithelial cells composing the BBB. This method perhaps represents the most promising avenue for targeted NIR-II imaging of the central nervous system. 

Not only can membrane encapsulation be applied toward the targeting of specific tissue types, but it can also be used for photoacoustic imaging. NIR-II-absorbing polypyrroles enveloped in cancer cell membranes demonstrated a guided PA imaging of tumors in the mouse model [138]. The NIR-II AIEgen developed by Cui et al. afforded a precise, targeted imaging of cancer cells, again using membrane camouflaging [139]. Rather than relying on leaky vasculature to mark cancerous tissue, CCNPs directly intermingle with homologous tissue types.

## 6. Conclusions

The development of next-generation NIR-II-sensitive imaging probes will require a detailed understanding of the latest nanobiotechnology. The brightness of a fluorescent molecule is determined by the product of its fluorescence quantum yield and its absorption cross-section at the excitation wavelength. Similarly, for ideal photoacoustic imaging (PAI) contrast agents, a large molar extinction coefficient, a sharp-peaked absorption spectrum, peak absorption in the SWIR window, and high photostability are desired. Emerging NIR-II-sensitive materials will be made to be targeted, stable, and safe for in vivo imaging. Functionalization technology opens possibilities to tailor imaging contrast agents for improved specificity, sensitivity, and performance in fluorescence and PA imaging modalities. The combinations of transport and functionalization technologies will be specific to the use case and tissue target. To date, NIR-II nanoprobes have been used in fluorescence and photoacoustic angiography, tumor imaging, the tracking of cellular migration, examining biological distribution and clearance pathways, tracking the location and differentiation of transplanted stem cells, the monitoring of collagen degradation, the imaging of inflammation in the brain and lymph nodes, and the monitoring of bacterial infections. NIR-II contrast agents will continue to be a platform for studying tissue structures and disease pathologies.

It is essential to acknowledge that NIR-II optical imaging technology is still in its early stages and faces some key challenges. One major concern is the limited stability of many organic NIR-II nanoprobes in water, restricting their clinical utility. Additionally, a comprehensive understanding of the biocompatibility, metabolic pathways, and long-term toxicity of NIR-II nanoprobes within the body is crucial, with many inorganic probes falling short in this regard. Furthermore, conventional NIR-II optical imaging systems suffer from drawbacks such as a narrow field of view and low resolution. These limitations must be overcome for the widespread use of NIR-II optical imaging in clinical settings.

As technology continues to evolve, the development of novel nanoprobes with improved photophysical properties, enhanced brightness, and better stability is expected. Researchers will likely focus on fine-tuning the design of NIR-II fluorophores and exploring various functionalization techniques to enable the targeted and specific imaging of disease biomarkers. Additionally, efforts to improve cytotoxicity evaluation and biocompatibility of imaging probes will be crucial for their successful translation into clinical settings. The integration of multimodal imaging approaches, by combining fluorescence and photoacoustic modalities, is anticipated to provide complementary information, enabling more accurate and comprehensive assessments of biological processes. Moreover, advancements in instrumentation and imaging systems will likely facilitate a higher spatial and temporal resolution, enabling real-time imaging and the dynamic monitoring of biological events at the molecular level. The future of NIR-II imaging probes in fluorescence and photoacoustic imaging is bright, with the potential to revolutionize non-invasive imaging in preclinical research. 

## Data Availability

No new data were created or analyzed in this study. Data sharing is not applicable to this article.
